# Decreased levels of endocytic collagen receptor Endo180 in dermal fibroblasts lead to decreased production of type I collagen and increased expression of matrix metalloproteinase‐1

**DOI:** 10.1111/phpp.12728

**Published:** 2021-09-09

**Authors:** Hiroyasu Iwahashi, Yoshihito Kawashima, Hitoshi Masaki

**Affiliations:** ^1^ Research Center Maruzen Pharmaceuticals Co. Ltd. Fukuyama City Japan; ^2^ Laboratory of Photoaging Research School of Bioscience and Biotechnology Tokyo University of Technology Hachioji City Japan

**Keywords:** collagen fragment, denatured collagen, Endo180, matrix metalloproteinase‐1, type I collagen

## Abstract

**Background:**

Endo180 is involved in collagen remodeling by incorporating extracellular degraded collagen. Ultraviolet irradiation of dermal fibroblasts reduces Endo180 expression, which affects collagen fiber remodeling. However, it is unclear whether the decrease in Endo180 is directly related to the decrease in type I collagen fibers during photoaging. We aimed to clarify the relationship between Endo180 reduction and the decrease in type I collagen fibers observed in photoaged dermis.

**Methods:**

Endo180 was reduced in normal human dermal fibroblasts using RNAi. Endo180 knockdown cells were inoculated into collagen gels. The influence of Endo180 knockdown was evaluated by measuring mRNA expression of collagen fiber remodeling‐related factors and collagen gel contraction. The collagen state and oxidative stress in the collagen gels were also measured.

**Results:**

Endo180 knockdown cells, which were confirmed by gelatin uptake inhibition, showed upregulation of matrix metalloproteinase‐1 and downregulation of type I collagen mRNA expression when cultured in collagen gels. The contractility of the collagen gel was reduced by Endo180 knockdown. The collagen state in the extracellular matrix of the collagen gels containing Endo180 knockdown fibroblasts showed increased amounts of 3/4 fragmented collagen and denatured collagen and decreased type I collagen synthesis. In addition, an increase in intracellular oxidative stress was observed.

**Conclusions:**

This study confirmed that the decrease in Endo180 caused a failure in collagen fiber formation and a decrease in collagen production, reproducing the photoaging dermal structural changes. This suggests that the decrease in Endo180 may be involved in wrinkle formation, which is a characteristic of photoaged skin.

## 1 INTRODUCTION

Long‐term ultraviolet (UV) exposure alters the dermal matrix architecture, resulting in a loss of collagen fibers and oxytalan fibers in the papillary dermis and the accumulation of large elastic fibers in the reticular dermis.^1^ Among dermal fibers, the loss of type I collagen fibers, which function as dermal skeletal fibers, has been demonstrated to play a crucial role in the progression of photoaging.^2^

Type I collagen fibers are maintained by the metabolic balance of synthesis and degradation, known as collagen fiber remodeling. The degradation process can be roughly divided into two processes: Extracellular cleavage of collagen fibers by matrix metalloproteinase (MMP) and internalization of the collagen degradation product into fibroblasts by Endo180, an endocytic collagen receptor.^3^

Endo180 (MRC2/uPARAP/CD280), a type‐1 membrane protein belonging to the mannose receptor family, is expressed on the plasma membranes of fibroblasts, macrophages, and endothelial cells.^4^, ^5^, ^6^

Endo180 has the ability to uptake at least types I, IV, and V collagen.^7^ Of these, type I collagen has been reported in in‐vitro studies to be a target for Endo180 binding and internalization, ranging from intact collagen to 3/4 and 1/4 fragmented collagen cleaved by MMP‐1 to denatured collagen (gelatin).^8^ However, it has been reported that the binding ability of these collagens to Endo180 is higher in flexible structures, such as denatured collagen, than in linear structures, such as intact collagen, and the binding site of Endo180 has also been determined by crystal structure analysis.^9^ It has been demonstrated that the collagen degradation products recognized by Endo180 are incorporated into endosomes by clathrin‐dependent endocytosis and degraded to amino acids by proteases in lysosomes.^10^

Recently, the accumulation of type I collagen 3/4 fragments and the loss of Endo180 have been observed in the dermis of chronic sun‐exposed elder subjects.^11^ In addition, a single exposure of the skin to solar‐simulated UV was shown to reduce Endo180 levels in fibroblasts. Furthermore, fibroblasts exposed to UVA show decreased Endo180 expression.^12^ Moreover, our previous study reported that interleukin‐1 alpha derived from UVB‐exposed keratinocytes induces the loss of Endo180 in fibroblasts, and we have discovered interaction between keratinocytes and fibroblasts in UVB‐induced Endo180 suppression.^13^

Although it is expected that the accumulation of collagen fragments, associated with a decrease in Endo180, impairs the regular remodeling of collagen fibers by inducing dysfunction of fibroblasts, thereby altering the environment surrounding the fibroblasts, the influence of the absence of Endo180 on collagen fiber remodeling is still unclear. Thus, this study was conducted to determine the influence of Endo180 reduction on the reconstruction of collagen and to clarify the role of Endo180 in the progression of photoaged skin.

## 2 MATERIALS AND METHODS

### 2.1 Cell culture

Normal human skin fibroblast cell line (NB1RGB) was purchased from RIKEN BioResource Research Center and cultured in Dulbecco's modified Eagle's medium (DMEM) (Nissui Pharmaceutical) containing 10% fetal bovine serum (FBS) (Biosera) and penicillin/streptomycin (Wako Pure Chemical Corporation) at 37°C in an atmosphere consisting of 5% CO_2_. Fibroblasts passaged nine to 11 times were used for following experiments.

### 2.2 RNA interference

Fibroblasts were inoculated into 6‐well plastic plates and cultured in DMEM containing 10% FBS without antibiotics for 1 day. A mixture of Lipofectamine 3000 (Invitrogen) and control siRNA or Endo180 siRNA (Santa Cruz Biotechnology) in serum and antibiotic‐free DMEM was added, and the cells were further cultured for 3 days. After harvesting with trypsin/EDTA, the cells were inoculated under each experimental condition. Knockdown efficiency was determined by mRNA and protein expression on day 3, as described below, and the gelatin internalization test was used to confirm loss of function.

### 2.3 Quantitative real‐time RT‐PCR

Total RNA was isolated from fibroblasts using ISOGEN II (Nippon Gene) in accordance with a standard operating method, and cDNAs were synthesized with PrimeScript RT Master Mix using TaKaRa PCR Thermal Cycler Dice Touch (TaKaRa Bio). Real‐time RT‐PCR for Endo180, COL1A1, MMP‐1, and GAPDH was performed with TB Green Fast qPCR Mix using Thermal Cycler Dice Real Time System III (TaKaRa Bio), and the primer sets were purchased from Takara Bio. The Endo180, COL1A1, and MMP‐1 mRNA expression was normalized to GAPDH expression and expressed as percentages against the expression in control cells.

### 2.4 Western blotting

Protein lysates of fibroblasts were prepared using the Mammalian Protein Extraction Reagent (Thermo Fisher Scientific). Equal amounts of protein lysates were subjected to sodium dodecyl sulfate‐polyacrylamide electrophoresis with 4%‐20% gradient gels (Bio‐Rad Laboratories) and transferred to polyvinylidene difluoride membranes using the Trans‐Blot Turbo Transfer System (Bio‐Rad Laboratories). After blocking with Block Ace (DS Pharma Biomedical), the membrane was incubated with the following primary antibodies: mouse anti‐human Endo180 antibody (Santa Cruz Biotechnology) or mouse anti‐GAPDH antibody (Abcam) for 2 hours at 25°C. After washing in phosphate‐buffered saline with 0.05% Tween‐20 (PBS‐T), the membrane was incubated with horseradish peroxidase‐conjugated anti‐mouse IgG antibody (Santa Cruz Biotechnology) for 1 hour at 25°C. After washing in PBS‐T, the blots were detected with the ECL‐PRIME (GE Healthcare) and ChemiDoc XRS systems (Bio‐Rad Laboratories). Endo180 production was expressed as a percentage of the level in control cells.

### 2.5 Gelatin internalization test

Fibroblasts were pretreated with 20 μmol/L EST (Merck), a lysosomal protease inhibitor, for 1 hour to prevent the degradation of internalized collagen. Cells were incubated with Oregon Green 488‐conjugated gelatin (OG‐gelatin, Invitrogen), which acted as the denatured collagen, at a concentration of 25 μg/mL for 2 hours. After washing, the cells were further incubated with 0.4% trypan blue for 5 minutes to quench any extracellular fluorescence from the gelatin.^14^ The cells were photographed using an Olympus fluorescence microscope.

### 2.6 Collagen gel preparation

Fibroblasts were suspended in type I atelocollagen gel medium at 1.0 × 10^5^ cells/mL using AteloCell IPC‐30 or 3D‐LG01 (KOKEN) at 1‐4 mg collagen/mL. The mixture was placed in 12‐well plastic plates or glass bottom cell culture slides, which were immediately warmed to 37°C to form a gel. After 1 hour of incubation, the gels were overlaid with a double volume of DMEM and incubated until used.

### 2.7 Collagen gel contraction assay

The gels were detached from the dishes prior to incubation for 3 days. Each gel area was measured using the ChemiDoc XRS system and Image Lab software (Bio‐Rad Laboratories).

### 2.8 Observation of collagen situation in collagen gel‐cultured fibroblasts

Collagen gel immunofluorescent staining was carried out following the methods reported by Bennink et al.^15^ Briefly, fibroblast‐embedded collagen gels cultured for 2 days were fixed with 10% formalin neutral buffer solution (Wako Pure Chemical Corporation) for 4 hours at 25°C. The fragmented collagen, denatured collagen, and collagen production from fibroblasts in collagen gels were stained at 4°C overnight with rabbit anti‐collagen type I (3/4 fragment) antibody (AdipoGen), 5‐carboxyfluorescein conjugate collagen hybridizing peptide (3Helix Inc), which specifically binds to denatured collagen strands, and rabbit anti‐human type I collagen antibody (Novotec), respectively. Subsequently, the primary antibodies, nuclei, and actin in gels were stained at 25°C for 3 hours with Alexa Fluor 488‐conjugated goat anti‐rabbit IgG (H&L), Hoechst33342, and Phalloidin‐iFluor 594 Conjugate (Cayman Chemical), respectively. Each fluorescence image was captured by a BZ‐X800 fluorescent microscope (Keyence) using an axial optical sectioning module, with a 20× objective at a distance of 10‐40 μm from the bottom cover glass.

### 2.9 Oxidative stress in collagen gel‐cultured fibroblasts

Fibroblast‐embedded collagen gels cultured for 2 days were stained with CellROX^®^ Oxidative Stress Reagents (Thermo Fisher Scientific) to visualize reactive oxygen species (ROS) and Hoechst33342 to visualize the nuclei, for 1 hour at 37°C. Fluorescence images were captured using a fluorescent microscope with a 100× objective.

### 2.10 Statistical analysis

All data are expressed as the mean ± standard error of the mean (SEM). Comparisons between two groups were performed using Student's two‐tailed unpaired *t* test. A *P*‐value of <.05 was considered statistically significant.

## 3 RESULTS

### 3.1 Confirmation of Endo180 knockdown

**FIGURE 1:**
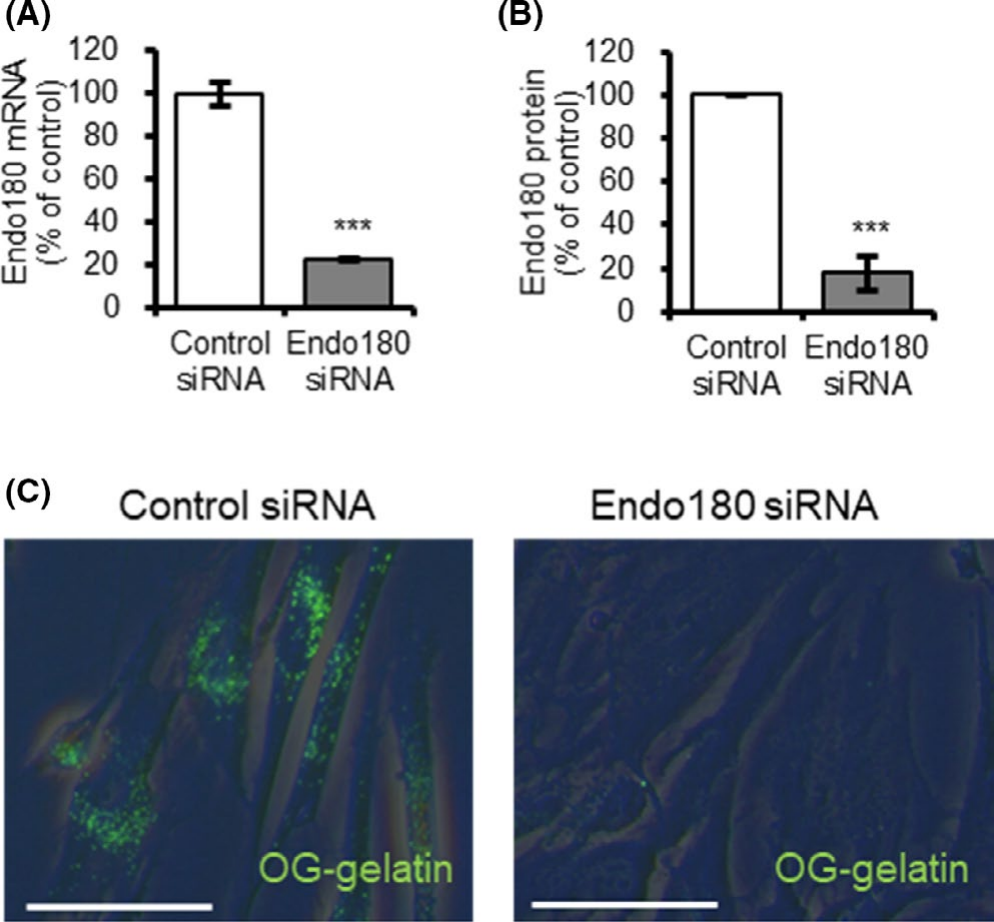
Influence of Endo180 knockdown on collagen internalization in human dermal fibroblasts. A–B, Endo180 mRNA expression and protein synthesis in cells cultured for 3 days after control or Endo180 siRNA transfection was examined by (A) qRT‐PCR and (B) western blotting. Bars indicate mean ± SE, n = 3, significance: ***P < .001. C, Collagen internalization in cells as in A–B after incubation with OG‐gelatin for 2 h was imaged by microscopy (Scale bar: 50 μm). Green fluorescence indicates internalized collagen [Colour figure can be viewed at wileyonlinelibrary.com]

When the gene expression was compared 3 days after the addition of control siRNA and Endo180 siRNA to dermal fibroblasts, Endo180 mRNA expression was reduced by 22% with the addition of Endo180 siRNA compared with the addition of control siRNA (Figure 1A). The expression at the protein level was also reduced by 18% (Figure 1B). In addition, the uptake of fluorescent gelatin almost completely disappeared in Endo180 siRNA‐loaded cells (Figure 1C).

### 3.2 Influence of Endo180 knockdown on gene expression in fibroblasts in collagen gel

**FIGURE 2:**
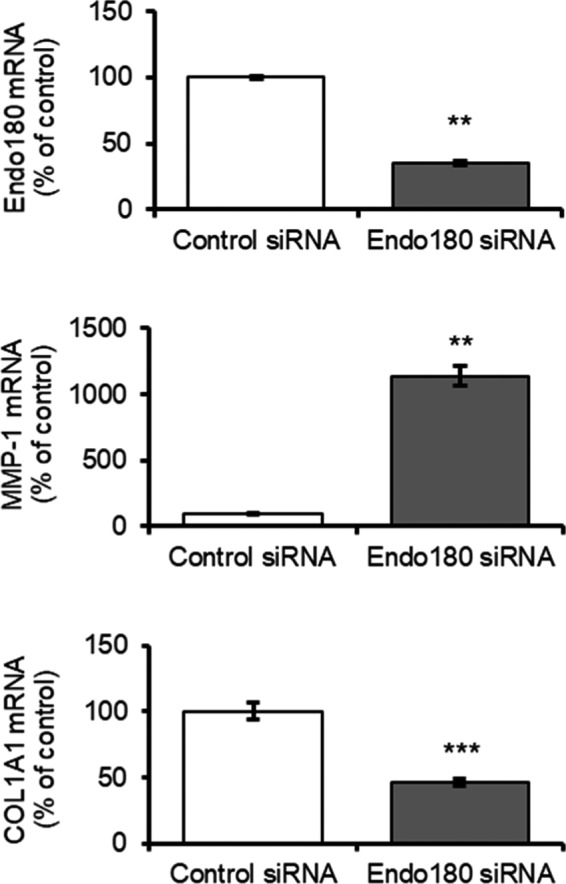
Influence of Endo180 knockdown on collagen synthesis and degradation in fibroblast‐embedded collagen gel. The cells were embedded into 3D collagen gel 3 days after control or Endo180 siRNA transfection. Endo180, MMP‐1, and COL1A1 mRNA expression in the cells at 3 days after embedding was examined by qRT‐PCR. Bars indicate mean ± SEM, n = 3, significance: **P < .01, ***P < .001

It was confirmed that Endo180 mRNA was decreased even on the second day after the cells were embedded in the gel, after 3 days from siRNA addition when Endo180 knockdown was confirmed. Under these conditions, the expression of MMP‐1 mRNA was markedly upregulated and that of COL1A1 mRNA was downregulated in Endo180 knockdown cells compared with the control cells (Figure 2).

### 3.3 Influence of Endo180 knockdown on collagen gel contraction

**FIGURE 3:**
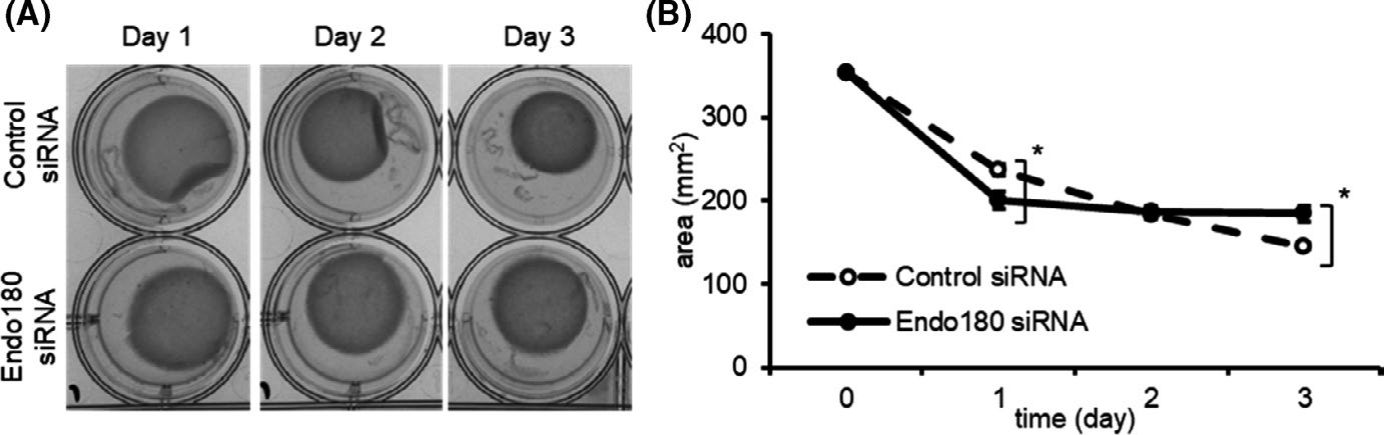
Influence of Endo180 knockdown on collagen gel contraction. The cells were embedded into 3D collagen gel 3 days after control or Endo180 siRNA transfection. The gels were detached from the dishes prior to incubation for 3 days. A, Representative collagen gel contraction images on each day taken using ChemiDoc system. B, The gel area was measured using Image Lab software. Data indicate mean ± SEM, n = 3, significance: *P < .05

To confirm the adverse effects of expression changes of genes that are important for collagen remodeling by Endo180 knockdown, gel contraction assay was performed. Collagen gel embedded with control siRNA‐treated fibroblasts showed continuous contraction after detachment, whereas collagen gel embedded with Endo180 knockdown fibroblasts shrank immediately after detachment and showed no further contraction for the next 2 days (Figure 3A). In other words, the area of the control cell‐embedded gel was larger than that of Endo180 knockdown cell‐embedded gel on day 1 and smaller than that of Endo180 knockdown cell‐embedded gel on day 3 (Figure 3B).

### 3.4 Influence of Endo180 knockdown on the collagen state in collagen gels

**FIGURE 4:**
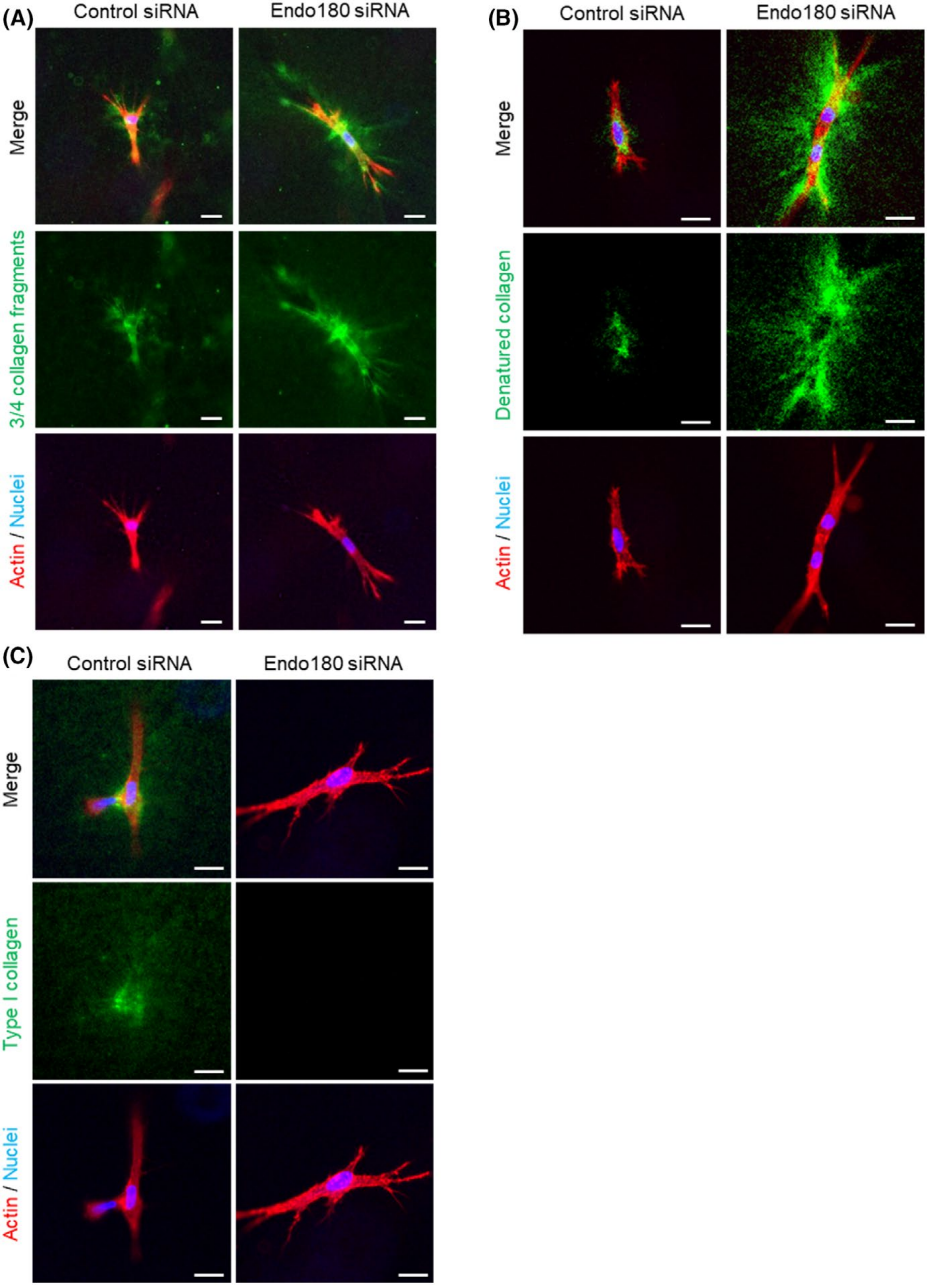
Influence of Endo180 knockdown on collagen synthesis and degradation in fibroblast‐embedded collagen gel. The cells were embedded into 3D collagen gel 3 days after control or Endo180 siRNA transfection. A–C, The gels at 2 days after embedding were fixed and stained with rabbit anti‐collagen type I (3/4 fragment) antibody and anti‐rabbit IgG antibody‐Alexa Fluor^®^ 488 to visualize the fragmented collagen (green) (A), 5‐FAM conjugate collagen hybridizing peptide to visualize the denatured collagen (green) (B), and rabbit anti‐human type I collagen antibody and anti‐rabbit IgG antibody‐Alexa Fluor^®^ 488 to visualize the collagen production (green) (C). The actin was stained with phalloidin‐iFluor™ 594 Conjugate (red), and the nuclei were counterstained with Hoechst33342 (blue). Representative images were taken using a fluorescent microscope with an axial optical sectioning module. Scale bar: 20 μm [Colour figure can be viewed at wileyonlinelibrary.com]

As abnormal gel contraction indicates the presence of adverse effects on collagen remodeling, we visualized fragmented collagen, denatured collagen, or newly produced type I collagen in the fibroblast‐embedded collagen gels to confirm the influence of Endo180 knockdown on collagen remodeling. Fluorescence derived from 3/4 fragmented collagen in collagen gels was observed around fibroblasts, and more 3/4 fragmented collagen was observed around Endo180 knockdown cells compared with control cells (Figure 4A). The denatured collagen in the collagen gel was scarcely observed around the control fibroblasts but mainly within the fibroblasts. However, a large amount of denatured collagen was observed around Endo180 knockdown fibroblasts (Figure 4B). In addition, although synthesis of type I collagen was observed around the control fibroblasts, newly synthesized type I collagen was not observed around the Endo180 knockdown fibroblasts (Figure 4C).

### 3.5 Influence of Endo180 knockdown on ROS in fibroblasts in collagen gel

**FIGURE 5:**
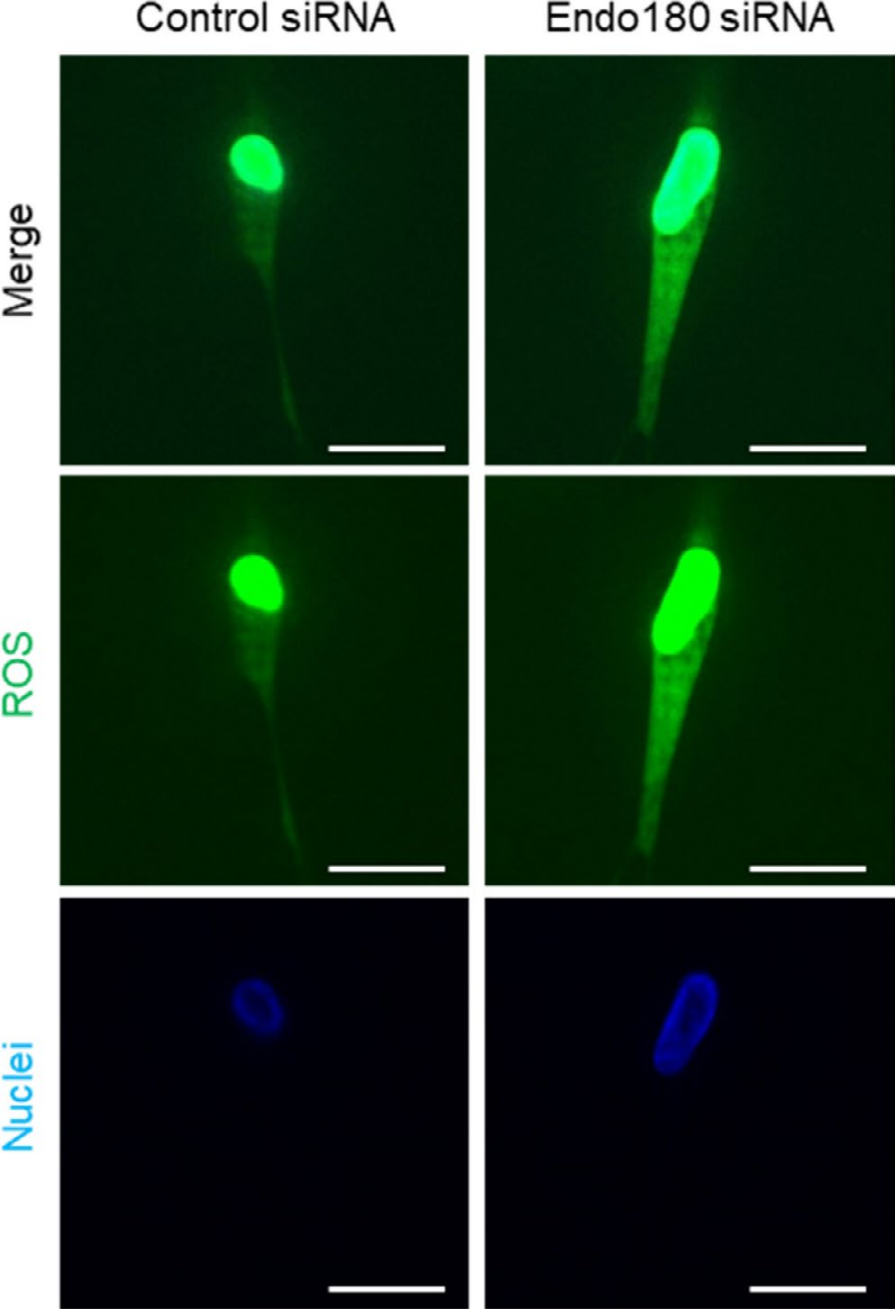
Influence of Endo180 knockdown on ROS generation and of collagen cleavage on collagen remodeling in fibroblast‐embedded collagen gel. The cells were embedded into 3D collagen gel 3 days after control or Endo180 siRNA transfection and incubated for 2 days. The gels were stained with CellROX^®^ Oxidative Stress Reagents to visualize ROS (green), and the nuclei were counterstained with Hoechst33342 (blue). Representative images were taken using a fluorescent microscope with an axial optical sectioning module. Scale bar: 20 μm [Colour figure can be viewed at wileyonlinelibrary.com]

To explore the cause of Endo180 knockdown‐induced changes in collagen remodeling‐related genes, we examined the generation of ROS in fibroblasts in collagen gels. A strong generation of ROS in the cytoplasmic region was observed in Endo180 knockdown fibroblasts compared with control fibroblasts (Figure 5).

## 4 DISCUSSION

In photoaged skin, the most prominent morphological changes in appearance, represented by wrinkles and sagging, are known to originate from architectural alterations in the dermal matrix, including changes to collagen fibers. Although collagen fibrils are normally maintained by a homeostatic balance of synthesis and degradation called collagen remodeling, it has been shown that Endo180, which is responsible for collagen uptake in collagen remodeling, is reduced by chronic exposure to UV light.^11^ This fact suggests that a decrease in Endo180 may inhibit collagen regeneration by decreasing the cellular uptake of degraded collagen fibers, resulting in a decrease in collagen fibers in photoaged skin. However, it is still unclear how the decrease in Endo180 affects the remodeling of collagen fibers and contributes to the progression of photoaged skin. Therefore, to investigate the contribution of Endo180 to the progression of photoaging, we investigated the behavior of Endo180 knockdown fibroblasts in collagen fiber remodeling using RNAi.

The creation of Endo180 knockdown fibroblasts by Endo180 siRNA treatment was confirmed by decreased Endo180 mRNA and protein expression and collagen internalization function (Figure 1). To date, it is unknown whether decreased Endo180 expression affects MMP‐1 expression. It has been reported that knockdown of Endo180 does not affect COL1A1 mRNA expression in monolayer cultured fibroblasts.^12^ Therefore, to mimic the dermal environment of fibroblasts more closely, Endo180 knockdown fibroblasts were embedded in collagen gels, and MMP‐1 and COL1A1 mRNA expression was evaluated. Surprisingly, there was a marked increase in MMP‐1 mRNA expression and a decrease in COL1A1 mRNA expression when cultured in collagen gel (Figure 2). Furthermore, Endo180 knockdown fibroblast‐embedded gels showed greater gel shrinkage than control fibroblast‐embedded gels at day 1, but no gel shrinkage was observed thereafter (Figure 3). In general, it is thought that the promotion of gel shrinkage represents an increase in fibroblast function, but it has also been reported that treatment of collagen gels with MMP‐1 also results in gel shrinkage.^16^ It has been confirmed that pro‐MMP‐1, produced as a precursor, is not activated in the fibroblast monolayer culture system. On the other hand, it has been reported that pro‐MMP‐1 produced by fibroblasts is activated in collagen gel cultures.^16^ These facts suggest that the contraction of Endo180 knockdown fibroblast‐embedded gel after 1 day was thought to originate from a decrease in the volume of the collagen gel itself due to the degradation of collagen fibers around the fibroblasts, caused by the activation of MMP‐1, whose production was enhanced by Endo180 knockdown. The subsequent cessation of contraction was thought to be due to a decrease in the interaction between fibroblasts and collagen fibers caused by environmental changes around the fibroblasts.

Based on the genetic changes caused by Endo180 knockdown and the abnormalities in gel contraction, it was thought that the collagen state in the gel was affected, and we confirmed that the collagen state around the fibroblasts was embedded in the collagen gel. As 3/4 fragmented collagen was observed in the pericellular region of the control fibroblasts and Endo180 knockdown fibroblasts (Figure 4A), it was suggested that MMP‐1 was activated in the collagen gel. In addition, fragmented collagen was frequently observed around Endo180 knockdown fibroblasts, suggesting that the degradation of collagen fibers was enhanced because of the increased production of MMP‐1. The ends of the collagen fragments degraded by MMP‐1 untwist the triple‐helical structure at body temperature because of the low denaturation temperature and denature into gelatinous form, which is further degraded by gelatinase.^17^, ^18^ Although 3/4 fragmented collagen was present around the control fibroblasts, the presence of denatured collagen was scarcely observed; however, it was observed in the cells (Figure 4B), suggesting that the denatured collagen was taken up by the fibroblasts immediately after denaturation. On the other hand, the accumulation of denatured collagen around Endo180 knockdown fibroblasts was observed, indicating that Endo180 knockdown fibroblasts were unable to take up the denatured collagen. This suggests that the gelatinous denatured collagen is taken up by fibroblasts via Endo180. Furthermore, the presence of new type I collagen was confirmed around the control fibroblasts but not around Endo180 knockdown fibroblasts, and the decrease in collagen production was associated with the decrease in COL1A1 mRNA expression, which was also confirmed at the protein level.

In addition, strong ROS production was observed in Endo180 knockdown cells embedded in the gel (Figure 5). Fisher et al^19^ reported excessive ROS production and increased MMP‐1 expression in fibroblasts cultured on fragmented collagen gels by treatment with MMP‐1. Furthermore, it has been postulated that this phenomenon is caused by a decrease in cellular tension resulting from fibroblast scaffold instability because of collagen fiber cleavage. In collagen remodeling, H_2_O_2_ or ^1^O_2_, which are types of ROS, increase MMP‐1 mRNA expression^20^, ^21^ and decrease COL1A1 mRNA expression.^22^ The mechanism involves increased levels of the transcription factor activated protein (AP)‐1 due to activation of EGF receptor signaling.^23^ AP‐1 upregulates mRNA expression of MMP‐1 and CYR61/CCN1, which is a negative feedback protein for collagen synthesis.^24^ Subsequently, CYR61 downregulates the mRNA expression of COL1A1.^25^ In the present study, increased production of MMP‐1 and the degradation of collagen fibers around the cells were observed in Endo180 knockdown fibroblasts embedded in collagen gels, which may replicate the fibroblast scaffold instability described by Fisher et al Therefore, one of the pathways by which Endo180 knockdown reduces collagen remodeling capacity is the accumulation of denatured collagen generated by MMP‐1‐induced degradation of collagen fibers around fibroblasts, which induces fibroblast scaffold instability. Additionally, it is thought that the increased ROS production caused the increased expression of MMP‐1 and a decrease in collagen synthesis.

The role of Endo180 in collagen remodeling during photoaged skin formation is described as follows: reduced expression of Endo180 caused by UV exposure leads to an accumulation of denatured collagen, which is derived from fragmented collagen produced by MMPs hyperproduced by UV exposure, around fibroblasts. As a result, the fibroblast scaffold becomes unstable, leading to the generation of excess ROS. ROS induces an increase in MMP‐1 production and a decrease in collagen production, which further aggravate the environment around the fibroblast, resulting in a negative loop of collagen remodeling.

In summary, a decrease in Endo180 induces the accumulation of denatured collagen around fibroblasts and maintains a reduced collagen remodeling capacity through increased oxidative stress. This study is the first to reveal the importance of the environment surrounding fibroblasts and the role of Endo180 in the formation of photoaged skin, albeit through an in‐vitro experiment. Although the presence of fragmented collagen fibers in the dermis of sun‐exposed areas has been reported, the presence of denatured collagen has not been confirmed. Thus, further studies will be conducted to clarify the presence of denatured collagen in sun‐exposed dermis and to determine the role of Endo180 in the formation of photoaged skin in vivo.

## CONFLICT OF INTEREST

The authors have no conflict of interest to declare.

## Data Availability

The data that support the findings of this study are available from the corresponding author upon reasonable request.
